# Physiotherapy interventions for the respiratory management of people with spinal cord injury: recommendations from an Australian and New Zealand clinical practice guideline

**DOI:** 10.1038/s41393-025-01116-7

**Published:** 2025-09-30

**Authors:** K. E. Tranter, L. A. Harvey, J. Ross, B. Wadsworth, D. J. Berlowitz, E. A. Bye, L. W. Chen, H. Patterson, M. McDonald, S. Calthorpe, J. Agostinello, E. Gollan, S. Denis, L. Blecher, D. Wilson, J. Peach, T. McDonald, M. Walters, J. Mather, L. Davis, M. D’Cruz, J. V. Glinsky

**Affiliations:** 1 The John Walsh Centre for Rehabilitation Research, University of Sydney, Sydney, NSW Australia; 2 Victorian Spinal Cord Service, Melbourne, VIC Australia; 3 Princess Alexandra Hospital, Metro South Health, Brisbane, QLD Australia; 4 Victorian Respiratory Support Service, Melbourne, VIC Australia; 5 Melbourne University, Melbourne, VIC Australia; 6 Neuroscience Research Australia, Sydney, NSW Australia; 7 University of New South Wales, Sydney, NSW Australia; 8 Royal North Shore Hospital, Sydney, NSW Australia; 9 The Alfred Trauma Service, Melbourne, VIC Australia; 10 Prince of Wales Hospital, Sydney, NSW Australia; 11 Spinal Outreach Team, Adelaide, SA Australia; 12 Hampstead Rehabilitation Centre, Adelaide, SA Australia; 13 Auckland Spinal Rehabilitation Unit, Counties Manukau, Health New Zealand, Auckland, New Zealand; 14 Middlemore hospital, Manukau District, Health New Zealand, Auckland, New Zealand; 15 Australian Quadriplegic Association, Sydney, NSW Australia

**Keywords:** Spinal cord diseases, Rehabilitation

## Abstract

**Study design:**

Development of respiratory recommendations in a Clinical Practice Guideline (CPG).

**Objectives:**

(i) To describe the evidence recommendations and consensus-based opinion statements for the effectiveness of respiratory interventions to improve respiratory muscle strength, lung volumes and secretion clearance. (ii) To outline the clinical rationale for these recommendations and statements.

**Setting:**

Australia and New Zealand

**Methods:**

Sixteen clinical questions relating to respiratory interventions for people with spinal cord injury (SCI) were presented in PICO format (Participant, Intervention, Comparison, Outcome) and decided a-priori by a respiratory guideline committee. Systematic reviews were conducted to answer each of the questions using rigorous methodology to synthesise evidence from randomised controlled trials. Evidence was assessed for risk of bias and quality using Grading of Recommendations Assessment Development and Evaluation (GRADE). Details were presented to a guideline panel who voted on each question and developed either an evidence recommendation or a consensus-based opinion statement.

**Results:**

Eighteen randomised controlled trials met the inclusion criteria and were relevant for eight questions. Three of these trials had interventions and outcomes relevant to more than one question. Overall, ten, eight and two trials answered questions related to improving respiratory muscle strength, lung volumes and secretion clearance, respectively.

**Conclusion:**

The Australian and New Zealand CPGs for the physiotherapy management of people with SCI (www.sciptguide.com) provide evidence recommendations and consensus-based opinion statements to inform respiratory management of people with SCI.

## Introduction

Respiratory muscle weakness occurs in people with spinal cord injury (SCI) with motor levels above T12 (as per the International Standards for Neurological Classification of SCI) [1]. The impact of respiratory muscle weakness is more pronounced in people with higher and more complete injuries. Respiratory muscle weakness leads to reduced lung volumes and a decreased ability to cough, therefore, increasing the susceptibility to hypoventilation, atelectasis and secretion retention. Overall, the effects of respiratory muscle weakness are lifelong and consequently can pose problems in both the acute period and long term after injury. As such, respiratory impairments are a leading cause of mortality following SCI [2, 3]. Physiotherapy interventions are commonly used both acutely and life-long to manage the respiratory impairments of people with SCI.

Common physiotherapy interventions used to manage respiratory impairments after SCI include respiratory muscle training [4–13], positioning [14], manual assisted cough, insufflation-exsufflation and abdominal binders [14–17]. Clinicians and people with SCI require resources that provide clear guidance about these interventions. There are existing resources to assist physiotherapists to understand respiratory impairments after SCI [18, 19]. There are also numerous randomised controlled trials (RCTs), systematic reviews and evidence summaries that examine the evidence base behind these treatments [18, 20–23]. However, there were no Clinical Practice Guidelines (CPGs) about the physiotherapy management of people with SCI that provided a synthesis of this evidence for clinicians. Guidelines are useful for clinicians because they use a rigorous process to interpret the evidence, summarise the available information and formulate recommendations.

This paper reports on the respiratory recommendations and statements from the Australian and New Zealand CPGs for the physiotherapy management of people with SCI [24]. This paper has two key objectives. First, to describe the evidence recommendations and consensus-based opinion statements for the effectiveness of respiratory interventions to improve respiratory muscle strength, lung volumes and secretion clearance in people with SCI. Second, to outline the clinical rationale for these recommendations and consensus statements.

## Methods

The methodology for The Australian and New Zealand CPGs for the physiotherapy management of people with SCI is summarised below and has been described in detail elsewhere [25]. A respiratory CPG development committee (known as a panel) of 21 people was formed to develop the respiratory component of the CPG. The panel decided a priori on 16 respiratory PICO (Participant, Intervention, Comparison, Outcome) questions (Table 1). These 16 questions are the focus of this paper.

**Table 1:** Summary of PICO questions with associated evidence recommendations or consensus-based opinion statements made by the guideline panel.

PICO Question	Recommendations and consensus statements
Respiratory muscle training (vs no intervention) on inspiratory respiratory muscle strength in people with SCI who have respiratory muscle weakness	Weak EVIDENCE recommendation FOR: Respiratory muscle training may be used to improve respiratory muscle strength in people with SCI who have respiratory muscle weakness.
Abdominal binders in sitting (vs no intervention) on lung volumes in people with SCI who have abdominal muscle weakness or paralysis	Weak EVIDENCE recommendation FOR: Abdominal binders in sitting may be used to improve lung volumes in people with SCI.
Supine (vs sitting) on lung volumes in people with SCI who have abdominal muscle weakness or paralysis	Strong CONSENSUS-BASED opinion statement FOR: Positioning in supine should be provided to improve lung volumes in people with SCI who have abdominal muscle weakness or paralysis.
Intermittent positive pressure (vs no intervention) on lung volumes in non- ventilated people with SCI who have respiratory muscle weakness	Strong CONSENSUS-BASED opinion statement FOR: Intermittent positive pressure should be provided to improve lung volumes in non-ventilated people with SCI who have respiratory muscle weakness.
Intermittent positive pressure (vs no intervention) on lung volumes in ventilated people with SCI who have respiratory muscle weakness	Strong CONSENSUS-BASED opinion statement FOR: Intermittent positive pressure should be used for improving lung volumes in ventilated people with acute SCI that are medically stable.
Deep breathing exercises (vs no intervention) on lung volumes in people with SCI who have respiratory muscle weakness	Weak CONSENSUS-BASED opinion statement FOR: Deep breathing exercises may be provided to improve lung volumes in people with SCI.
Air stacking (vs no intervention) on lung volumes in people with SCI who have respiratory muscle weakness	Weak CONSENSUS-BASED opinion statement FOR: Air stacking may be taught to improve lung volume in people with SCI who have respiratory muscle weakness.
Abdominal FES (vs no intervention) on lung volumes in people with SCI who have respiratory muscle weakness	NO evidence recommendation or consensus-based opinion statement: There is no recommendation for the use of abdominal FES to improve lung volumes in people with SCI who have respiratory muscle weakness.
Postural drainage (vs no intervention) on secretion clearance in people with SCI who have respiratory muscle weakness or paralysis	Strong CONSENSUS-BASED opinion statement FOR: Targeted postural drainage should be provided to improve secretion clearance in people with SCI who have respiratory muscle weakness or paralysis.
Manually assisted cough (vs no intervention) on secretion clearance in people with SCI who have abdominal muscle weakness or paralysis and an ineffective cough	Strong CONSENSUS-BASED opinion statement FOR: Manually assisted cough should be provided to improve secretion clearance in people with SCI who have abdominal muscle weakness or paralysis and an ineffective cough.
Mechanically assisted cough (Insufflation/exsufflation) (vs no intervention) on secretion clearance in people with SCI who have abdominal muscle weakness or paralysis and an ineffective cough	Strong CONSENSUS-BASED opinion statement FOR: Mechanically assisted cough (insufflation-exsufflation) should be provided to improve secretion clearance in people with SCI who have abdominal muscle weakness or paralysis and an ineffective cough.
Mechanically assisted cough (Insufflation/exsufflation) plus manually assisted cough (vs no intervention) on secretion clearance in people with SCI who have abdominal muscle weakness or paralysis and an ineffective cough	Strong CONSENSUS-BASED opinion statement FOR: A combination of mechanically assisted cough (insufflation-exsufflation) and manually assisted cough should be provided to improve secretion clearance in people with SCI who have abdominal muscle weakness or paralysis and an ineffective cough.
Percussion and vibration (vs no intervention) on secretion clearance in people with SCI who have respiratory muscle weakness	Weak CONSENSUS-BASED opinion statement FOR: Percussion and vibrations may be provided to improve secretion clearance in people with SCI who have respiratory muscle weakness.
Abdominal FES (vs no intervention) on stimulated cough in people with SCI who have abdominal muscle weakness or paralysis	Weak CONSENSUS-BASED opinion statement FOR: FES to the abdominal muscles may be provided to improve stimulated cough in people with SCI who have abdominal muscle paralysis or weakness.
Abdominal binders (vs no intervention) to improve cough in people with SCI who have abdominal muscle weakness or paralysis	Weak CONSENSUS-BASED opinion statement FOR: An abdominal binder may be provided to improve cough in people with SCI who have abdominal muscle paralysis or weakness.
Positive expiratory pressure devices (vs no intervention) on secretion clearance in people with SCI who have expiratory muscle weakness	Weak CONSENSUS-BASED opinion statement AGAINST: Positive expiratory pressure devices should not be provided to improve secretion clearance in people with SCI who have expiratory muscle weakness.

*SCI* spinal cord injury, *FES* functional electrical stimulation.

### Systematic reviews of the evidence

Systematic reviews were conducted for each PICO to synthesise available evidence from randomised controlled trials (RCTs). Each review aimed to determine the effectiveness of each respiratory intervention compared with no intervention or a sham intervention on outcomes for three impairments: respiratory muscle strength, lung volume or secretion clearance. Databases were searched from inception to August 2020 using a search strategy for RCTs combined with terms for SCI. We searched Ovid EMBASE, Ovid MEDLINE, EBSCO CINAHL Plus, Physiotherapy Evidence Database (PEDro) and the Cochrane Central register of controlled trials. The current published CPGs include summaries of RCTs up until August 2020 [24]. This paper has extended this search beyond this to April 2024. Only one further RCT investigating the effectiveness of a physiotherapy intervention on respiratory muscle strength was found [26]. This new study will be considered in the next iteration of the Guidelines.

### Risk of bias and GRADE

Risk of bias was assessed for each study using the Risk of Bias 2 (RoB 2) [27] and PEDro [28] quality assessment tools. The RoB 2 tool was used as part of the Grading of Recommendations Assessment, Development and Evaluation (GRADE) methodology [29]. GRADE methodology was used to determine the certainty of the evidence. Outcomes for each question were assessed using GRADE’s four-point scale (high certainty, moderate certainty, low certainty or very low certainty).

### Voting process and decisions about evidence recommendations and consensus-based statements

A panel with expertise in the respiratory management of people with SCI voted on recommendations and consensus statements. They were provided with a summary of findings and were trained in key aspects of the Evidence to Decision (EtD) Framework [30]. The panel considered the EtD criteria when making recommendations: the priority of the problem, benefits and harms of the intervention, certainty of the evidence, values and preferences of relevant stakeholders, resource utilisation, equity, acceptability and feasibility of intervention delivery. The panel first voted as to whether it was possible to make an evidence recommendation. This required 75% agreement within three rounds of voting. Panel members then voted in one of four ways. They voted strongly or weakly in favour of an intervention, or strongly or weakly against an intervention.

The guideline panel went to a consensus-based opinion statement instead of an evidence recommendation in the following situations: no RCT, a single study that provided inconclusive results or two trials that gave conflicting estimates of treatment effects that were of low or very low certainty as per GRADE. For consensus-based opinion statements panel members again voted in one of four ways. That is, strongly or weakly in favour of an intervention, or strongly or weakly against an intervention. The hierarchy of evidence recommendations and consensus-based opinion statements are described in Table 2.

**Table 2:** Summary of the strength of evidence recommendations and consensus-based opinion statements.

Evidence Recommendation	Explanation
Strong evidence recommendation FOR	The guideline panel is confident that they can recommend the intervention based on the evidence. A recommendation is made that the intervention should be implemented.
Weak evidence recommendation FOR	The guideline panel is confident that they can probably recommend the intervention based on the evidence. A recommendation is made that the intervention may be implemented.
Weak evidence recommendation AGAINST	The guideline panel is confident that they probably cannot recommend the intervention based on the evidence. A recommendation is made that the intervention should not be implemented.
Strong evidence recommendation AGAINST	The guideline panel is confident that they cannot recommend the intervention based on the evidence. A recommendation is made that the intervention should definitely not be implemented.
No recommendation	The guideline panel is unable to recommend for or against the intervention based on the evidence. A consensus-based opinion statement is made.

Consensus-based opinion statements	Explanation
Strong consensus FOR	The guideline panel is confident that they can recommend the intervention based on opinion. A statement is made that the intervention should be implemented.
Weak consensus FOR	The guideline panel is confident that they can probably recommend the intervention based on opinion. A statement is made that the intervention may be implemented.
Weak consensus AGAINST	The guideline panel is confident that they probably cannot recommend the intervention based on opinion. A statement is made that the intervention should not be implemented.
Strong consensus AGAINST	The guideline panel is confident that they cannot recommend the intervention based on opinion. A statement is made that the intervention should definitely not be implemented.
No consensus	The guideline panel is unable to make a statement for or against the intervention based on opinion.

Evidence recommendations are based on the GRADE approach.

## Results

Eighteen RCTs were used to inform the 16 questions related to respiratory interventions (Table 3). Six of these were randomised cross over trials. Three trials had interventions and outcomes that were relevant to more than one of the questions [14, 15, 31]. Ten, eight and two trials answered questions related to improving respiratory muscle strength, lung volumes and secretion clearance respectively. The GRADE rating was applied to eight questions all of which were judged as very low certainty (Table 4).

**Table 3:** Characteristics of included trials.

Study	Comparisons	Dosage	ROB 2/PEDro score	Design	Outcome	N (Rx/C)	Participants
**PICO:** Respiratory muscle training (v no intervention) on inspiratory respiratory muscle strength in people with SCI who have respiratory muscle weakness							
Boswell-Ruys et al. [9]	• Respiratory muscle training • Sham	3–5 sets 12 breaths 2 x day 5 days per week for 6 weeks @ > 30% MIP	Very low Risk of Bias PEDro = 10/10	Between-subject	Maximal Inspiratory Pressure (MIP)	29/31	C4–C8 SCI AIS A,B,C
Liaw et al. [11]	• Inspiratory muscle training & usual care • Usual care	15–20 min 2 x day; 7 days per week for 6 weeks	High Risk of Bias PEDro = 4/10	Between-subject	Maximal Inspiratory Pressure (MIP)	10/10	C4–C7 complete SCI <6months post injury
Litchke et al. [33]	• Respiratory resistance training • No intervention	1 set of exercises 2–3 x per day daily for 10 weeks	Some Concerns about Risk of Bias PEDro = 5/10	Between-subject	Maximal Inspiratory Pressure (MIP)	4/5	>80% participants with SCI C5–T12 SCI >6months post injury
Litchke et al. [34]	• Concurrent flow resistance • No intervention	10 breaths 3 different x per day daily for 9 weeks	High Risk of Bias PEDro = 3/10	Between-subject	Maximal Inspiratory Pressure (MIP)	5/7	>80% participants with SCI C5–C7 SCI
Loveridge et al. [4]	• Inspiratory muscle training • No intervention	85% of sustained inspiratory pressure 2 x day for 15 min 5 days per week for 8 weeks	Some Concerns about Risk of Bias PEDro = 4/10	Between-subject	Maximal Inspiratory Pressure (MIP)	6/6	C6–C7 complete SCI > 1 year post injury
Mueller et al. [6]	• Inspiratory resistance training • Placebo	90 breaths @ > 80% max inspiratory power 4 x per week for 8 weeks	High Risk of Bias PEDro = 5/10	Between-subject	Maximal Inspiratory Pressure (MIP)	8/8	C5–C8 complete SCI 6–8 months post injury
Postma et al. [12]	• Resistive inspiratory muscle training & usual care • Usual care	7 sets of 2 min @ 60% MIP; 5 x week for 8 weeks	High Risk of Bias PEDro = 7/10	Between-subject	Maximal Inspiratory Pressure (MIP)	19/21	T12 and above SCI AIS A-D; initial rehab; FEV_1_ < 80% predicted
Roth et al. [5]	• Expiratory muscle training • Sham	10 reps, twice a day, 5 x per week for 6 weeks	High Risk of Bias PEDro = 4/10	Between-subject	Maximal Inspiratory Pressure (MIP)	16/13	T1 and above motor complete SCI
Soumyashree et al. [8]	• Inspiratory muscle training • Breathing exercises^a^	15 min @ 40% MIP; 5 x per week for 4 weeks	Some Concerns of Risk of bias PEDro = 7/10	Between-subject	Maximal Inspiratory Pressure (MIP)	15/12	T1–12 SCI; AIS A-D
West et al. [7]	• Inspiratory muscle training • Sham	30 breaths at 50–60% Pi_max_ 2 x day; 5 days per week for 6 weeks	High Risk of Bias PEDro = 4/10	Between-subject	Maximal Inspiratory Pressure (MIP)	5/5	C5–C7 SCI AIS A or B ≥3years post-injury
Sikka et al. [26]^b^	• RIMT • Control	3 × 12 inspirations, 3 × 12 expirations 2 x day 5 days per week for 4 weeks @ > 30% MIP (40 supervised sessions)	High Risk of Bias PEDro = 3/10	Between-subject	Maximal Inspiratory Pressure (MIP)	48/48	C4–C7 AIS A, B
**PICO:** Abdominal binders in sitting (v no intervention) on lung volumes in people with SCI who have abdominal muscle weakness or paralysis							
Boaventura et al. [14]	• Sitting with abdominal binder • Sitting without abdominal binder	N/A	Some Concerns of Risk of bias PEDro = 6/10	Within-subject	Forced Vital Capacity (FVC)	10/10	C4–C7 Complete SCI; >1 year
Bodin et al. [35]	• Sitting with abdominal binder • Sitting without abdominal binder	N/A	High Risk of Bias PEDro = 4/10	Within-subject	Vital Capacity (VC)	20/20	C5–C8 SCI; > 1 year
Goldman et al. [16]	• Sitting with abdominal binder • Sitting without abdominal binder	N/A	High Risk of Bias PEDro = 5/10	Within-subject	Vital Capacity (VC)	7/7	C5–C7 complete SCI; >3 months post injury
Hart et al. [17]	• Sitting with abdominal binder • Sitting without abdominal binder	N/A	High Risk of Bias PEDro = 4/10	Within-subject	Forced Vital Capacity (FVC)	10/10	C5–T6 AIS A SCI
Wadsworth et al. [15]	• Sitting with abdominal binder • Sitting without abdominal binder	N/A	High Risk of Bias PEDro = 4/10	Within-subject	Forced Vital Capacity (FVC) / Peak Expiratory Flow (PEF)	14/14	C3–T5 AIS A or AIS B SCI Acute
**PICO:** Supine (v high sitting) on lung volumes in people with SCI who have abdominal muscle paralysis or weakness.							
Boaventura et al. [14]	• Sitting with abdominal binder • Sitting without abdominal binder	N/A	Some Concerns of Risk of bias PEDro = 6/10	Within-subject	Forced Vital Capacity (FVC)	10/10	C4–C7 Complete SCI; >1 year
**PICO:** Intermittent positive pressure (v no intervention) on lung volumes in non- ventilated people with SCI who have respiratory muscle weakness							
Laffont et al. [36]	• Intermittent positive pressure breathing (IPPB) • No Intervention	IPPB up to 40 cmH_2_0; 20 mins 2 x per day; 5 days per week for 2 months	High Risk of Bias PEDro = 5/10	Within-subject	Vital Capacity (VC)	14/14	C5–T6 Complete SCI < 6 months post injury
**PICO:** Air stacking (v no intervention) on lung volumes in people with SCI who have respiratory muscle weakness							
Jeong et al. [38]	• Air stacking • Incentive spirometry^a^	20 reps air stacking 2 x per day; 5 days per week for 6 weeks	High Risk of Bias PEDro = 6/10	Between-subject	Forced Vital Capacity (FVC)	14/12	Tetraplegia
**PICO:** Abdominal FES (v no intervention) on lung volumes in people with SCI who have respiratory muscle weakness							
Cheng et al. [31]	• NMES plus usual care • Usual care	NMES 30 Hz; pulse width 300 µs; on/off 4/4 s; Intensity 0–100 mA.	Some concerns about Risk of Bias PEDro = 5/10	Between-subject	Forced Vital Capacity (FVC)	13/13	C4–C7 SCI; AIS A, B; <3 months post injury
**PICO:** Abdominal FES (v no intervention) on stimulated cough in people with SCI who have abdominal muscle weakness or paralysis							
Cheng et al. [31]	• NMES plus usual care • Usual care	NMES 30 Hz; pulse width 300 µs; on/off 4/4 s; Intensity 0–100 mA.	Some concerns about Risk of Bias PEDro = 5/10	Between-subject	Peak Expiratory Flow (PEF)	13/13	C4–C7 SCI; AIS A, B; <3 months post injury
**PICO:** Abdominal binders (v no intervention) to improve cough in people with SCI who have abdominal muscle weakness or paralysis							
Wadsworth et al. [15]	• Sitting with abdominal binder • Sitting without abdominal binder	N/A	High Risk of Bias PEDro = 4/10	Within-subject	Forced Vital Capacity (FVC) / Peak Expiratory Flow (PEF)	14/14	C3–T5 AIS A or AIS B SCI Acute

Comparison refers to the two groups included in this review; Dosage refers to the amount of therapy provided to the experimental group; N refers to the number of participants who contributed to the analysis (not necessarily the number of participants randomised).

*RIMT* respiratory inspiratory muscle training, *MIP* maximal inspiratory pressure, *PEDro* physiotherapy evidence database, *AIS* American spinal injury association (ASIA) impairment scale as per the international standards for neurological classification of spinal cord injury (ISNCSCI), *NMES* neuromuscular electrical stimulation, *FEV*_*1*_ forced expiratory volume in 1 s.

^a^This was determined to be equivalent to sham intervention.

^b^Trial wasn’t included in meta-analysis as found in the most recent literature search (September 2020 to April 2024). This trial is to be included in future revisions of the Guidelines.

**Table 4:** GRADE rating of outcomes for each PICO question where randomised controlled trials were available.

PICO Question	GRADE	RATING				
		Risk of bias	Inconsistency	Imprecision	Indirectness	Publication Bias
Respiratory muscle training (v no intervention) on inspiratory respiratory muscle strength in people with SCI who have respiratory muscle weakness	Very low certainty	Serious	Serious	Not serious	Not serious	Serious
Abdominal binders in sitting (v no intervention) on lung volumes in people with SCI who have abdominal muscle weakness or paralysis	Very low certainty	Very serious	Not serious	Not serious	Serious	Serious
Supine (v high sitting) on lung volumes in people with SCI who have abdominal muscle weakness or paralysis	Very low certainty	Very serious	Serious	Serious	Serious	Serious
Intermittent positive pressure (v no intervention) on lung volumes in non- ventilated people with SCI who have respiratory muscle weakness	Very low certainty	Very serious	Serious	Serious	Serious	Serious
Air stacking (v no intervention) on lung volumes in people with SCI who have respiratory muscle weakness	Very low certainty	Very Serious	Serious	Serious	Serious	Serious
Abdominal FES (v no intervention) on lung volumes in people with SCI who have respiratory muscle weakness	Very low certainty	Very serious	Not serious	Serious	Not serious	Serious
Abdominal FES (v no intervention) on stimulated cough in people with SCI who have abdominal muscle weakness or paralysis	Very low certainty	Serious	Serious	Not serious	Not serious	Serious
Abdominal binders (v no intervention) to improve cough in people with SCI who have abdominal muscle weakness or paralysis	Very low certainty	Serious	Serious	Not serious	Not serious	Serious

**Fig. 1:**
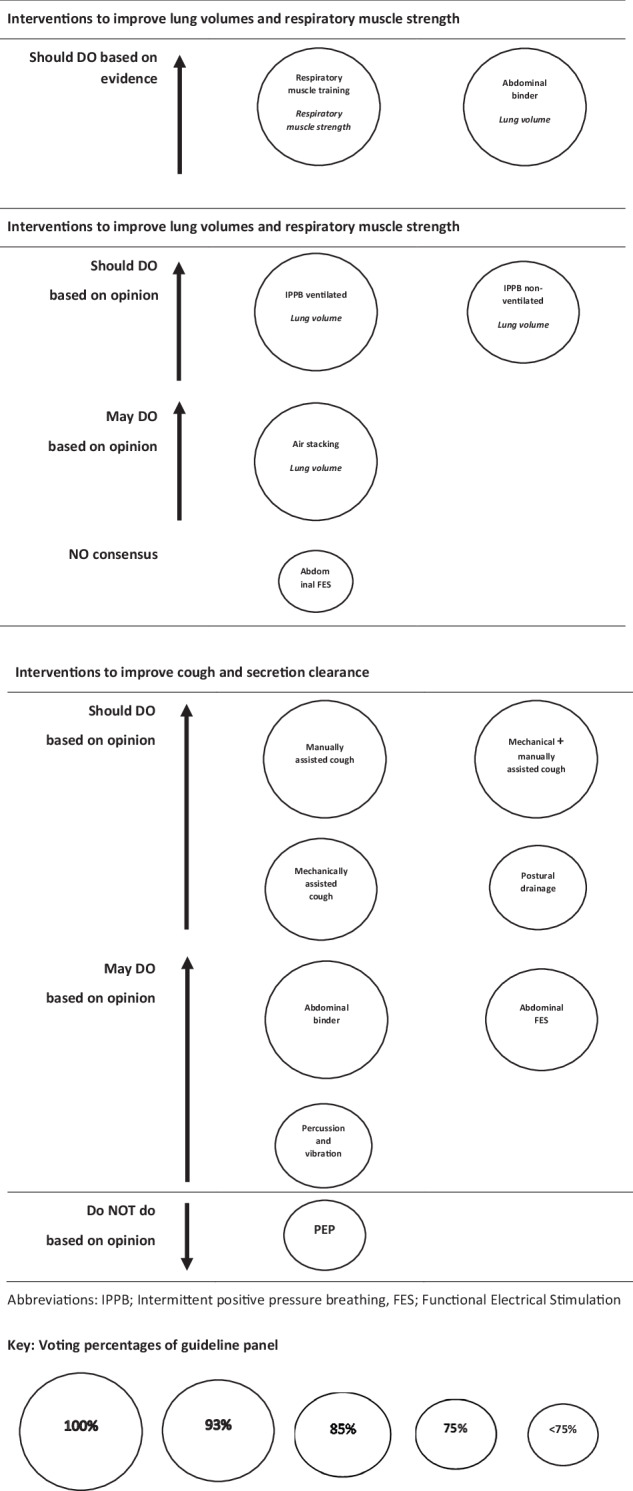
Bubble plot summarising strength of evidence recommendations and consensus-based opinion statements of interventions for managing people with SCI and respiratory impairment. The size of the circle denotes the percentage of the panel that voted for the recommendation.

The panel developed two evidence recommendations and 13 consensus-based opinion statements. There was one question where no evidence recommendation or consensus-based opinion statement could be made. In addition, three statements were made about the overall principles of management. Details of the evidence recommendations and consensus-based opinion statements for each question are provided below and summarised in Table 1 and Fig. 1.

### Overall principles of respiratory physiotherapy management

The panel voted on three consensus statements that encompassed overall principles of respiratory physiotherapy management. They were:

1. People with newly acquired SCI with respiratory muscle weakness should be assessed by a physiotherapist within 24 h of admission to hospital.
2. People with existing SCI admitted for the management of a respiratory condition should be assessed by a physiotherapist within 24 h of admission to hospital.
3. People with SCI and respiratory muscle weakness who are at a high risk of respiratory complications should be discharged from hospital into the community with a respiratory management plan in place (including education to the care team on appropriate interventions).

### Physiotherapy interventions for respiratory muscle strength

#### Respiratory muscle training vs no intervention on respiratory muscle strength

**Fig. 2:**
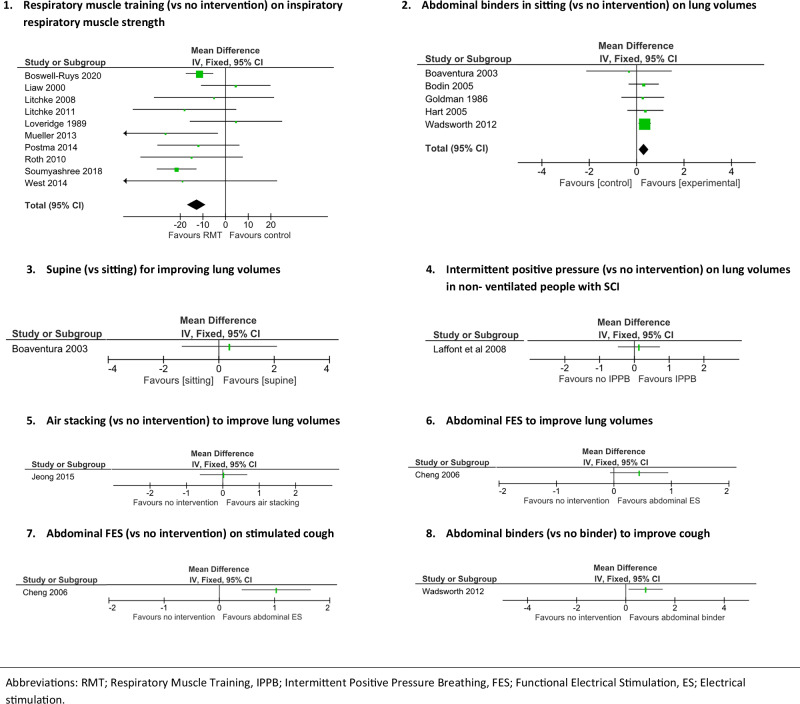
Meta-analysis and forest plots for respiratory PICOs.

A weak evidence recommendation for respiratory muscle training to improve respiratory muscle strength in people who have respiratory muscle weakness was formed by considering the results of ten RCTs [4–9, 11, 12, 32, 33]. The pooled results of the meta-analysis indicated that respiratory muscle training is better than no respiratory muscle training (mean between group difference (95% CI) in maximal inspiratory pressure was −13 cmH_2_0 (−17 to −9); see Fig. 2) [24]. This recommendation was formed considering the results of the meta-analysis, the very low certainty of evidence (as per GRADE) as well as the EtD criteria such as resource use, cost effectiveness and the acceptability and feasibility of delivering the intervention.

##### Clinical rationale

Respiratory muscle training commonly refers to both inspiratory (IMT) and expiratory muscle training. Inspiratory Muscle Training is most frequently used in clinical practice and involves breathing in against resistance to strengthen or improve the endurance of inspiratory muscles. It aims to strengthen skeletal muscles of inspiration, therefore needs to follow principles of progressive resistance training [34]. That is, it needs to be delivered with sufficient resistance and progressed as tolerated [9]. IMT is commonly delivered via a threshold or flow device which aims to increase the user’s inspiratory effort. People with acute or chronic conditions are likely to benefit, therefore IMT should be considered in hospital and community-based settings [9].

### Physiotherapy interventions for lung volumes

#### Abdominal binders vs no intervention on lung volumes

A weak evidence recommendation for abdominal binders to improve lung volumes in people who have abdominal muscle weakness or paralysis was formed by considering the results of five RCTs [14–17, 35]. The pooled results of the meta-analysis indicated that abdominal binders in sitting are better than no abdominal binders for improving lung volumes (mean between group difference (95% CI) in lung volumes was 0.3 litres (0.1 to 0.5); see Fig. 2). This recommendation was formed considering the results of the meta-analysis, the very low certainty of evidence (as per GRADE) as well as the EtD criteria such as resource use, cost effectiveness and the acceptability and feasibility of delivering the intervention.

##### Clinical rationale

Abdominal binders are used in people that have abdominal weakness and respiratory impairments. The binder aims to improve intra-abdominal pressure by reversing the increase in abdominal compliance associated with abdominal weakness. This in turn optimises the position of the diaphragm by elevating it, improving its length tension relationship and hence ability to contract, increasing the amount of air drawn into the lungs [18]. The abdominal binder is only effective for improving lung volumes when in-situ. There are no long-term effects when the binder is removed. Abdominal binders may not be suitable for people with significant abdominal distension or large abdomens.

#### Positioning supine vs sitting on lung volumes

A strong *c*onsensus-based opinion statement for positioning in supine versus (vs) sitting to improve lung volumes in people who have abdominal muscle paralysis or weakness was formed by considering the results of one RCT [14] (Fig. 2). The results of this RCT could not inform an evidence recommendation.

##### Clinical rationale

The supine position allows optimal positioning of the diaphragm to generate larger inspiratory volumes for people with SCI and abdominal weakness or paralysis. That is, when someone with abdominal weakness or paralysis sits up their abdomen splays outwards and their diaphragm flattens. In this position the diaphragm is unable to effectively contract to generate the same inspiratory volumes compared to if the person was supine [18]. As such, improved lung volumes are best achieved while the person is supine. The supine position may not be suitable for people with significant abdominal distension / central adiposity. In this instance, it may be necessary to put the person in a head-up tilt position.

#### Intermittent application of positive pressure device vs no intervention on lung volumes in non-ventilated people

A strong consensus-based opinion statement for the intermittent application of positive pressure devices to improve lung volumes in non-ventilated people with acute SCI who have respiratory muscle weakness was formed by considering the results of one RCT [36] (Fig. 2). The results of this RCT could not inform an evidence recommendation.

##### Clinical rationale

Positive pressure devices include mechanical insufflation, Intermittent Positive Pressure Breathing (IPPB), Continuous Positive Airway Pressure (CPAP) and brief periods of Bilevel Positive Airway Pressure (BiPAP). They all aim to hyperinflate the lungs to increase tidal volume and minute ventilation and improve the volume of gas exchanged [19]. Positive pressure can be used as an adjunct to other physiotherapy techniques and can be applied immediately before, during and / or immediately post treatment to optimise lung volumes or reduce the work of breathing. It can be applied during physiotherapy treatment to target ventilation in a specific patient position or to increase inspiratory volumes and hence expiratory volumes and flow to augment secretion removal. The use of positive pressure is contraindicated in conditions that include but are not limited to untreated pneumothorax, tracheoesophageal fistula and acute traumatic brain injury with increased or poorly controlled intracranial pressure.

#### Intermittent application of positive pressure vs no intervention on lung volumes in ventilated people

A strong consensus-based opinion statement for intermittent application of positive pressure to improve lung volumes in ventilated people with acute SCI that are medically stable was formed based on the opinion of the guideline panel (as there were no RCTs on this PICO).

##### Clinical rationale

Ventilator hyperinflation is the preferred option in ventilated patients to avoid disconnection from the ventilator and loss of Positive End Expiratory Pressure [37]. The use of positive pressure is contraindicated in conditions that include but are not limited to untreated pneumothorax, tracheoesophageal fistula and acute traumatic brain injury with increased or poorly controlled intracranial pressure. Positive pressure in ventilated patients is performed in consultation with medical staff.

#### Deep breathing exercises vs no intervention on lung volumes

A weak consensus-based opinion statement for deep breathing exercises to improve lung volumes was formed based on the opinion of the guideline panel (as there were no RCTs on this PICO).

##### Clinical rationale

Deep breathing requires increased voluntary effort of the inspiratory muscles to generate greater inspiratory volumes above resting tidal volumes. Deep breathing exercises will be of most benefit to people with SCI who have sufficient respiratory muscle strength to take a deep breath [18].

#### Air stacking vs no intervention on lung volumes

A weak consensus based-opinion statement for air stacking to improve lung volumes in people with SCI who have respiratory muscle weakness was formed by considering the results of one RCT [38] (Fig. 2). The results of the RCT could not inform an evidence recommendation.

##### Clinical rationale

Air stacking involves repetitive inspiratory breaths at intervals, stacking one breath on top of the other. Inspiratory lung volumes are gradually increased with repetitive inhalations using a positive pressure inspiratory device. It is commonly provided by a manual resuscitator bag via a mouthpiece (and nose peg) [38]. A mouthpiece (with a nose peg) is used rather than a face mask because facemasks increase the risk of pneumothorax. Air stacking should not be used in any person with associated contraindication(s) to positive pressure including but not limited to untreated pneumothorax, tracheoesophageal fistula and acute traumatic brain injury with increased or poorly controlled intracranial pressure.

#### Abdominal functional electrical stimulation vs no intervention on lung volumes

No evidence recommendation or consensus-based opinion statement was formed for the use of abdominal Functional Electrical Stimulation (FES) to improve lung volumes in people with SCI who have respiratory muscle weakness. One RCT for this PICO was considered, however, the results of the RCT could not inform an evidence recommendation [31] (Fig. 2) and the guideline panel did not reach consensus.

##### Clinical rationale

Abdominal FES involves the application of surface stimulation to the abdominals to contract the weak or paralysed abdominal muscles [39]. Abdominal FES is more commonly used to improve cough effectiveness than lung volumes.

### Physiotherapy interventions for cough and secretion clearance

#### Postural drainage vs no intervention on secretion clearance

A strong consensus-based opinion statement for postural drainage to improve secretion clearance in people with SCI who have respiratory muscle weakness or paralysis was formed based on the opinion of the guideline panel (as there were no RCTs on this PICO).

##### Clinical rationale

Postural drainage involves positioning the patient to target a specific lung segment to allow gravity to act to drain secretions to the more central airways [40]. Postural drainage (including head down tilt) is usually provided as an adjunct to other respiratory therapies (such as intermittent positive pressure; percussions and vibrations); or forced expiratory techniques (e.g., cough, manual assist cough +/− insufflation/exsufflation, insufflation/exsufflation). Head down tilt is contraindicated in conditions that include but are not limited to heart failure, reflux and acute traumatic brain injury with increased/poorly controlled intracranial pressure.

#### Manually assisted cough vs no intervention on secretion clearance

A strong consensus-based opinion statement for manually assisted cough to improve secretion clearance in people with SCI who have abdominal muscle weakness or paralysis, and an ineffective cough was formed based on the opinion of the guideline panel (as there were no RCTs on this PICO).

##### Clinical rationale

Manually assisted cough involves the application of external pressure to the abdomen and/or anterior thorax. It is a common technique used to clear secretions in someone with respiratory muscle weakness and can be applied by one or two therapists with the patient in supine or sitting [19]. It compensates for weak abdominal and intercostal muscles by providing abdominal +/− thoracic over pressure in conjunction with a voluntary cough (to ensure closure of the glottis and the build-up of intrabdominal and intrathoracic pressure) to increase peak expiratory flow rates [41]. Manually assisted cough is contraindicated in people with recent abdominal trauma. It should be considered with caution in people with paralytic ileus, abdominal distension, rib fractures, obesity and who have recently eaten.

#### Mechanically assisted cough (insufflation/exsufflation) vs no intervention on secretion clearance

A strong consensus-based opinion statement for mechanically assisted cough to improve secretion clearance in people with SCI who have abdominal muscle weakness or paralysis, and an ineffective cough was formed based on the opinion of the guideline panel (as there were no RCTs on this PICO).

##### Clinical rationale

A mechanically assisted cough involves the application of positive pressure to augment lung volumes (insufflation) followed by a rapid shift to negative pressure (exsufflation) to augment the movement of secretions to the central airways where secretions can be cleared via a cough or removed via suction. A mechanically assisted cough can be applied via a face mask, mouthpiece or tracheostomy to augment a patient’s cough, thereby improving treatment efficiency and reducing patient fatigue and potential need for invasive suction [42]. Contraindications and precautions for the use of positive pressure must be considered before prescribing mechanically assisted cough such as untreated pneumothorax, tracheoesophageal fistula and acute traumatic brain injury with increased/poorly controlled intracranial pressure.

#### Mechanically assisted cough (insufflation/exsufflation) plus manually assisted cough vs no intervention on secretion clearance

A strong consensus-based opinion statement for a combination of mechanically assisted cough and manually assisted cough to improve secretion clearance in people with SCI who have abdominal muscle weakness or paralysis, and an ineffective cough was formed based on the opinion of the guideline panel (as there were no RCTs on this PICO).

##### Clinical rationale

The combination of manual plus mechanically assisted cough may be used clinically. The clinical rationale, contraindications and precautions for manually assisted cough and mechanically assisted cough have been provided above.

#### Percussion and vibration vs no intervention on secretion clearance

A weak consensus-based opinion statement for percussion and vibrations to improve secretion clearance in people with SCI who have respiratory muscle weakness was formed based on the opinion of the guideline panel (as there were no RCTs on this PICO).

##### Clinical rationale

Percussion and vibration transmit mechanical forces through the airways to mobilise secretions. Percussions involve the application of rhythmical tapping with a cupped hand, usually over targeted areas of the lungs [40]. Vibrations involve shaking of the chest wall, usually on expiration, with the direction of force following the natural bucket handle motion of the chest wall. Both percussions and vibrations are provided with other respiratory interventions such as postural drainage, mechanical insufflation-exsufflation, intermittent positive pressure therapies and other secretion clearance techniques.

#### Abdominal FES vs no intervention on stimulated cough

A weak consensus-based opinion statement for FES to the abdominal muscles to improve stimulated cough in people with SCI who have abdominal muscle weakness or paralysis was formed by considering the results of one RCT [31] (Fig. 2). The results of the RCT could not inform an evidence recommendation because there was only one small low-quality trial.

##### Clinical rationale

Abdominal FES involves the application of surface stimulation to the abdominals to contract the weak or paralysed muscles [39]. The contraction of the abdominal muscles in conjunction with a voluntary cough (to ensure closure of the glottis and the build-up of intrabdominal and intrathoracic pressure) can be used to augment expiratory flow rates.

#### Abdominal binder vs no intervention on cough

A weak consensus-based opinion statement for an abdominal binder to improve cough in people with SCI who have abdominal muscle weakness or paralysis was formed by considering the results of one RCT [15] (Fig. 2). The results of the RCT could not inform an evidence recommendation because there was only one small low-quality trial.

##### Clinical rationale

An abdominal binder is used to splint weak or paralysed abdominal muscles. In doing so, the abdominal binder prevents the splaying of the abdomen outwards and aims to keep the diaphragm in its optimal position. By ensuring the diaphragm is in its optimal position, it is able to effectively contract to generate larger inspiratory pressures and volumes. Consequently, increasing expiratory volumes and flow which may augment cough effectiveness [43]. Abdominal binders may not be suitable for people with significant abdominal distension or large abdomens.

#### Positive expiratory pressure devices vs no intervention on secretion clearance

A weak consensus-based opinion statement against positive expiratory pressure (PEP) for improving secretion clearance was formed based on the opinion of the guideline panel (as there were no RCTs on this PICO). The guideline panel was of the opinion that PEP should not be used to improve secretion clearance in people with SCI who have expiratory muscle weakness.

##### Clinical rationale

PEP is a common airway clearance technique achieved by breathing out into an external device. The external device provides resistance to the expiratory flow generating positive pressure throughout expiration. Common PEP devices include the Acapella® and Flutter®. PEP relies on sufficient expiratory flow rates to aid effective secretion clearance [40]. People with SCI and marked weakness or paralysis of the expiratory muscles rarely have sufficient expiratory flow rates to effectively clear secretions, hence PEP is not recommended.

## Conclusion

The respiratory component of the Australian and New Zealand CPG for the Physiotherapy Management of people with SCI provides recommendations and statements to guide respiratory management of people with SCI. These recommendations and statements are based on a rigorous methodology which considers RCTs amongst other factors. The hierarchy of recommendations is a useful tool for clinicians to consider when applying the recommendations and statements in practice. To support the recommendations and statements made in the Guidelines, this paper outlines the clinical rationale for the interventions described. These CPGs will support physiotherapists in their clinical decision making and increase consistency of care to all people with SCI that require respiratory management.

## Data Availability

Correspondence and request for materials should be addressed to KE Tranter keira.tranter@sydney.edu.au.
